# Work-Related Productivity Loss and Associated Indirect Costs in Patients With Crohn’s Disease or Ulcerative Colitis in the United States

**DOI:** 10.1093/crocol/otac023

**Published:** 2022-06-15

**Authors:** Zhijie Ding, Erik Muser, James Izanec, Rina Lukanova, James Kershaw, Adam Roughley

**Affiliations:** Janssen Scientific Affairs, LLC, Horsham, Pennsylvania, USA; Janssen Scientific Affairs, LLC, Horsham, Pennsylvania, USA; Janssen Scientific Affairs, LLC, Horsham, Pennsylvania, USA; Adelphi Real World, Bollington, UK; Adelphi Real World, Bollington, UK; Adelphi Real World, Bollington, UK

**Keywords:** inflammatory bowel disease, Crohn’s disease, ulcerative colitis, indirect costs, Work Productivity and Activity Impairment, presenteeism, absenteeism

## Abstract

**Background:**

Inflammatory bowel disease (IBD), including Crohn’s disease (CD) and ulcerative colitis (UC), affects an estimated 1.6 million US adults, and results in humanistic and economic burden even among mild patients, which grows with increasing disease activity.

**Methods:**

Gastroenterologists and their IBD patients provided real-world data via US IBD Disease Specific Programmes 2014–2018. Patients with physician- and patient-reported data completing a Work Productivity and Activity Impairment questionnaire were categorized by disease activity, defined using Crohn’s Disease Activity Index (CD) and partial Mayo scores (UC), respectively. Associations of disease activity with patient-reported productivity loss and indirect costs were assessed.

**Results:**

The analyses included 281 patients with CD and 282 patients with UC. Mean ages were 40.0 and 40.5 years, and mean disease durations 7.1 and 5.4 years, for CD and UC, respectively. In CD, absenteeism (0.95%–14.6%), presenteeism (11.7%–44.9%), and overall work impairment (12.4%–51.0%) increased with increasing disease activity (all *P* < .0001). In UC, absenteeism (0.6%–11.9%), presenteeism (7.1%–37.1%), and overall work impairment (7.5%–41.9%) increased with increasing disease activity (all *P* < .0001). Annual indirect costs due to total work impairment increased with increasing disease activity (all *P* < .0001), from $7169/patient/year (remission) to $29 524/patient/year (moderately-to-severely active disease) in CD and $4348/patient/year (remission) to $24 283/patient/year (moderately-to-severely active disease) in UC.

**Conclusions:**

CD and UC patients experienced increased absenteeism, presenteeism, and overall work impairment with increasing disease activity, resulting in higher indirect costs. Treatments significantly reducing IBD disease activity could provide meaningful improvements in work productivity and associated costs.

## Introduction

Inflammatory bowel disease (IBD), which includes Crohn’s disease (CD) and ulcerative colitis (UC), is a chronic autoimmune condition involving inflammation of the gastrointestinal tract.^1^ The Crohn’s and Colitis Foundation of America reported an estimated 1.6 million Americans having IBD in 2014.^2^ Recent epidemiological studies suggest the prevalence of IBD increased from 1990 to 2017 both in the United States and globally.^3^

Symptoms vary from patient to patient, but common symptoms associated with IBD include diarrhea, abdominal pain, rectal bleeding, anemia, fatigue, anxiety, and depression.^4,5^ While diarrhea and abdominal pain are the most frequently reported symptoms,^4^ fatigue has also been recognized as a burdensome problem, that is more pronounced in patients with active CD or UC.^6^ These symptoms have a dramatic impact on patient health-related quality of life (HRQoL), which is substantially impaired relative to the general population.^7^ IBD is often diagnosed in the second or third decade of life, and generally follows a long-term fluctuating disease course,^4^ which further contributes to the substantial impact on patients’ HRQoL.

The lack of a pharmacologic or surgical cure for CD and UC, as well as the frequent occurrence of relapses, contributes to considerable healthcare resource utilization and cost.^8^ While direct costs include the costs of medication, hospitalizations, and surgery,^9–12^ the humanistic burden of IBD, with symptoms such as fatigue and depression, also result in high indirect costs, due to impacts on employment and impaired productivity.^11–15^

An analysis of a national US survey showed an excess unemployment rate of 12.3% in patients with IBD who had experienced symptoms in the past 12 months, which led to more than $3.6 billion in indirect cost attributable to IBD in 1999.^13^ Annual indirect costs due to absenteeism and presenteeism, measured with the Work Productivity and Activity Impairment questionnaire (WPAI), were calculated to be significantly higher for patients with IBD in remission compared with a control population in a prospective study at a US tertiary IBD center in 2013–2014, at $17 766 versus $9179, respectively.^14^

The development of a range of advanced treatments over the past 20 years^16^ has made the achievement of remission a realistic treatment target for many patients with IBD.^17^ Given the potential for maintenance of reduced disease activity over a prolonged period with the appropriate therapeutic approach, consideration of the impact of a lower level of disease activity on the humanistic and economic burden of IBD is of interest. The objective of this study was therefore to provide contemporary real-world estimates of the work-related productivity loss and associated indirect costs for patients diagnosed with CD or UC in the United States. The association of disease activity with productivity loss and associated indirect costs was also assessed.

## Methods

### Study Design

Data were drawn from 2 Adelphi IBD Disease Specific Programme (DSP) survey waves conducted from November 2014 to March 2015 and from September 2017 to January 2018 in the United States, merged to provide a single large sample. DSPs are large, multinational, independent, and retrospective point-in-time surveys of physicians and their patients presenting in a real-world clinical setting, that describe current disease management, disease-burden impact, and associated treatment effects (clinical and physician perceived).

Physicians were instructed to complete a record form for their next 8 CD and 7 UC consecutively consulting patients who visited the physician for routine care, to mitigate against selection bias. This physician-reported questionnaire contained detailed questions on diagnosis, disease management, treatment history, clinical outcomes, and gastroenterologists’ level of satisfaction with control. Completion of the physician-reported questionnaire was undertaken through consultation of existing patient clinical records, as well as the judgment and diagnostic skills of the respondent physician, which is entirely consistent with decisions made in routine clinical practice.

Each patient for whom the physician completed a form was then invited to voluntarily complete a patient-reported questionnaire, and upon agreement provided their informed consent to participate. Patient-reported questionnaires included demographic and clinical data, disease history, treatment satisfaction assessment, and WPAI to assess the impact of IBD on their functioning.^18^ Patient-reported questionnaires were completed by the patient on the day of physician consultation, independently from their physician and were returned in a sealed envelope, ensuring the patient’s responses were kept confidential from their physician.

Physician participation was financially incentivized, with reimbursement upon survey completion according to fair market research rates. Patients were not compensated for participation.

A complete description of the methods of the survey has been previously published.^19^ Using a check box, patients provided informed consent for use of their anonymized and aggregated data for research and publication in scientific journals. Data were collected in such a way that patients and physicians could not be identified directly; all data were aggregated and deidentified before receipt.

### Participating Physicians and Patients

A geographically diverse sample of gastroenterologists in the United States were recruited to participate in the DSP. Physicians were identified by local field agents, and were eligible to participate in this survey if they were personally responsible for treatment decisions and management of patients with IBD.

Patients were eligible for inclusion if they were ≥18 years of age, had a physician-confirmed diagnosis of CD or UC, and visited their physician during the survey period. In addition to these criteria, patients with UC were required to have one or more of the following: a history of moderate-to-severe disease, be currently receiving or having ever received a corticosteroid, immunomodulator or biologic, or have had a full Mayo score >4 at some point in the course of their disease (there are no patients in this dataset that have never been moderate-to-severe, only ever received a 5-amino salicylic acid [5-ASA], and had a Mayo score of >4). These additional criteria were included due to the likelihood of UC patients having mildly active disease for a much longer period of time than CD patients; there were no additional criteria for CD patients.

The survey was fielded during 2 periods, with each iteration independent of the other, and the data from the surveys combined to provide a larger sample for analysis. It should be noted that the survey was designed to facilitate understanding of real-world clinical practice, and thus physicians only reported data they had access to at the time of the consultation. This therefore represents the evidence physicians had when making treatment and other clinical management decisions at that consultation. No tests, treatments, or investigations were performed as part of this survey.

### Variables

The WPAI questionnaire is a validated instrument to measure impairments in work and activities and has been used frequently in studies of IBD patients.^20,21^ The WPAI questionnaire generates percentages (0%–100%) relating to the last week of work undertaken by patients, quantifying absenteeism (percentage of time missed from work), presenteeism (percentage impairment while at work), overall work impairment (percentage due to absenteeism and presenteeism), and total activity impairment (percentage limitation in daily activities outside of work), with higher values indicating greater limitation.^18^ The partial Mayo (pMayo) score consists of the sum of scores for stool frequency, rectal bleeding, and physician’s global assessment, each scored on a 4-point scale from 0 to 3, with higher scores indicating greater disease activity.^22^ The Crohn’s Disease Activity Index (CDAI) score was derived from information provided via physician- and patient-reported questionnaires while the pMayo score was derived solely from physician-reported questionnaires. For patients with CD, disease activity categories were: CDAI score 0–150, remission; CDAI score 151–220, mild disease; CDAI score >220, moderate-to-severe disease. For patients with UC, disease activity categories were: pMayo score 0–1, remission; pMayo score 2–4, mild disease; pMayo score >4, moderate-to-severe disease.

### Analysis

The primary analysis included only patients who were working, had completed the WPAI questionnaire, and for whom matched physician- and patient-reported questionnaires were available, so that productivity findings could be examined by disease activity level. Demographics and clinical characteristics were compared across patients with different levels of disease activity. Patient-reported work productivity loss and associated indirect costs were assessed by disease activity. As a secondary analysis, reported in Supplementary Materials, demographics and clinical characteristics were compared between patients who were working (full- or part-time) and those who were not.

Patients were stratified by their current disease activity, based on CDAI score for patients with CD^23^ and the current pMayo score for patients with UC.^22^

The human capital method was used to calculate the indirect cost of IBD from rates of absenteeism, presenteeism, and overall work impairment and their related monetary costs, based on lost earnings.^24^ A mean individual income of $57 900 was used, based on the US Census Bureau’s Survey of Social and Economic data, using reported income in 2019,^25^ and was divided by 2080 (the average number of work hours in a year; 40 hours a week for 52 weeks in a year) to calculate an hourly rate. The percentage of absenteeism reported in the WPAI, based on the individuals’ work hours missed in the last week, was multiplied by the hourly rate and then scaled accordingly to provide annual costs for lost productivity. A similar approach was taken for the percentage of impairment while at work in the last week (presenteeism) reported in the WPAI and for overall work impairment.

Descriptive statistics were produced with mean, SD, and range reported for continuous variables, and frequency and percentage of patients falling into each category reported for categorical variables. Statistical tests were performed across activity levels and between working and nonworking patients using a Mann–Whitney or Kruskal–Wallis test for continuous variables and Fisher’s exact or chi-squared test for categorical variables. The significance level for all analyses was set at 5% and all tests were 2 sided. Missing data were not imputed; therefore, the sample size of patients could vary from variable to variable and is reported separately for each analysis.

### Ethical Approval

All questionnaires used in the IBD DSP(s) were reviewed by an ethics review board; the 2017 materials were reviewed and approved by the Western Institutional Review Board study exempt under the criteria 45 CFR §46.101(b)(2) (protocol number: AG8199), and the 2015 materials were reviewed and approved by the Freiburger Ethik-Kommission International (FEKI) (protocol number: 017/1679).

Data collection was undertaken in line with European Pharmaceutical Marketing Research Association guidelines.^26^ Each survey was performed in full accordance with relevant legislation at the time of data collection, including the US Health Insurance Portability and Accountability Act 1996,^27^ and Health Information Technology for Economic and Clinical Health Act legislation.^27^

## Results

### Study Participants

Data were collected from 215 gastroenterologists, who completed questionnaires on 1600 patients with CD and 1389 patients with UC. Matched physician- and patient-reported questionnaires, where the patient had completed the information allowing us to calculate CDAI and pMayo score, were available for 468 CD patients and 609 UC patients, respectively. The relatively low number of patients with all data required for the primary analyses was expected, as completion of the patient-reported questionnaire was voluntary.

### Demographics and Clinical Characteristics

#### Crohn’s disease

**Figure 1.**
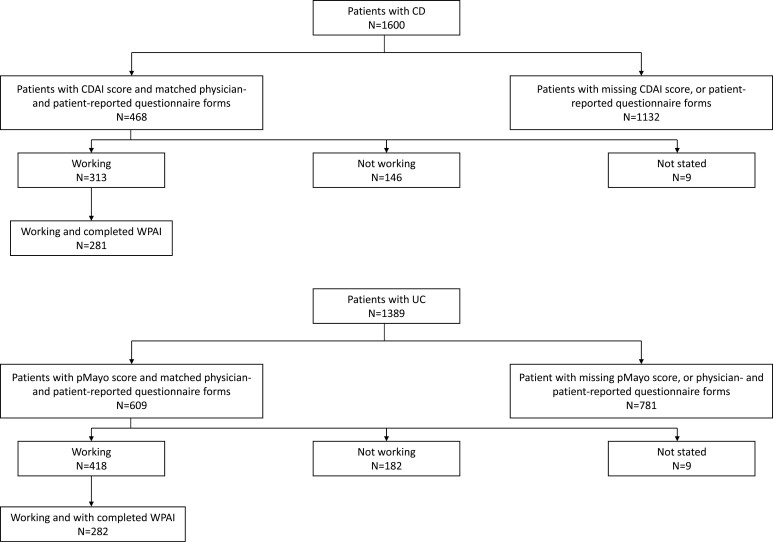
Disposition of patients from study population. Abbreviations: CD, Crohn’s disease; CDAI, Crohn’s Disease Activity Index; pMayo, partial Mayo score; UC, ulcerative colitis; WPAI, Work Productivity and Activity Impairment questionnaire.

A total of 281 patients with CD who had matched physician- and patient-reported questionnaires available, who were working, and who had completed the WPAI questionnaire were included in the primary analyses (Figure 1). Disease activity groups based on current CDAI score showed 183 (65.1%) patients were in remission, 59 (21.0%) had mildly active disease and 39 (13.9%) had moderately-to-severely active disease (Table 1). For patients classified as having moderately-to-severely active disease, the median CDAI score was 246, only 2 patients had a CDAI score >300 and none had a CDAI score >450.

**Table 1.** Demographics, clinical characteristics, and current treatments by CDAI-defined disease activity in patients with CD.

	Overall (*n* = 281)	Remission^a^ (*n* = 183)	Mild^a^ (*n* = 59)	Moderate-to-severe^a^ (*n* = 39)	*P*
Age, years					
*n*	281	183	59	39	
Mean (SD)	40.0 (12.1)	40.2 (11.9)	39.9 (13.0)	39.1 (12.1)	.8757
Range	18, 72	20, 66	18, 72	20, 64	
Gender, *n* (%)					
*n*	281	183	59	39	
Female	134 (47.7)	84 (45.9)	32 (54.2)	18 (46.2)	.5259
BMI, kg m^−2^					
*n*	281	183	59	39	
Mean (SD)	25.7 (4.1)	25.9 (4.1)	25.5 (3.9)	24.9 (4.6)	.2303
Range	16.2, 46.9	16.2, 46.9	18.1, 41.8	17.9, 42.4	
CDAI score					
*n*	281	183	59	39	
Mean (SD)	118.2 (80.6)	69.1 (46.2)	182.1 (20.2)	252.1 (25.6)	<.0001
Median	112	63	182	246	
Range	0, 324	0, 149	150, 220	222, 324	
Disease duration, years					
*n*	252	163	53	36	
Mean (SD)	7.1 (7.2)	7.5 (7.3)	6.8 (7.6)	5.6 (6.2)	.1037
Range	0, 40	0, 40	0, 33	0, 25	
Physician-reported patient ethnicity, *n* (%)					
*n*	281	183	59	39	
White/Caucasian	233 (82.9)	151 (82.5)	47 (79.7)	35 (89.7)	
Asian-Indian subcontinent	6 (2.1)	4 (2.2)	2 (3.4)	0 (0.0)	
Asian other	3 (1.1)	1 (0.5)	1 (1.7)	1 (2.6)	
Hispanic/Latino	6 (2.1)	4 (2.2)	1 (1.7)	1 (2.6)	.8220
Middle Eastern	2 (0.7)	1 (0.5)	0 (0.0)	1 (2.6)	
Mixed race	3 (1.1)	2 (1.1)	1 (1.7)	0 (0.0)	
African American	27 (9.6)	19 (10.4)	7 (11.9)	1 (2.6)	
Other	1 (0.4)	1 (0.5)	0 (0.0)	0 (0.0)	
Physician-reported current remission status^b^, *n* (%)					
*n*	281	183	59	39	
Not in remission	112 (39.9)	54 (29.5)	29 (49.2)	29 (74.4)	
In remission	145 (51.6)	106 (57.9)	30 (50.8)	9 (23.1)	<.0001
In deep/clinical remission	24 (8.5)	23 (12.6)	0 (0.0)	1 (2.6)	
Physician-reported current disease progression, *n* (%)					
*n*	281	183	59	39	
Improving	86 (30.6)	47 (25.7)	23 (39.0)	16 (41.0)	
Stable	176 (62.6)	130 (71.0)	31 (52.5)	15 (38.5)	.4909
Deteriorating	19 (6.8)	6 (3.3)	5 (8.5)	8 (20.5)	
Physician reported: patient currently experiencing a flare, *n* (%)					
*n*	251	169	50	32	
Yes	31 (12.4)	13 (7.7)	9 (18.0)	9 (28.1)	.0022
Physician reported: patient flared in past 12 months, *n* (%)					
*n*	251	169	50	32	
Yes	142 (56.6)	76 (45.0)	37 (74.0)	29 (90.6)	<.0001
Physician reported: number of flares/patient in the last 12 months					
*n*	251	169	50	32	
Mean (SD)	1.2 (1.9)	0.7 (1.3)	1.8 (2.1)	2.7 (2.8)	<.0001
Range	0, 16	0, 9	0, 9	0, 16	
Current treatment^c^, *n* (%)					
*n*	273	180	55	38	
Biologic	168 (61.5)	113 (62.8)	32 (58.2)	23 (60.5)	.8208
5-ASA	113 (41.4)	70 (38.9)	25 (45.5)	18 (47.4)	.4969
Immunomodulator	89 (32.6)	52 (28.9)	20 (36.4)	17 (44.7)	.1333
Corticosteroid	52 (19.0)	21 (11.7)	20 (36.4)	11 (28.9)	<.0001
Patient-reported satisfaction with current treatment, *n* (%)					
*n*	277	179	59	39	
Satisfied	201 (72.6)	155 (86.6)	30 (50.8)	16 (41.0)	<.0001
Not satisfied	76 (27.4)	24 (13.4)	29 (49.2)	23 (59.0)	

Abbreviations: 5-ASA, 5-amino salicylic acid; BMI, body mass index; CD, Crohn’s disease; CDAI, Crohn’s Disease Activity Index. *n* values are reported per row for extra clarity, as the different variables used in analyses do not always have the same sample sizes.

CDAI score 0–150, remission; CDAI score 151–220, mild disease; CDAI score >220, moderate-to-severe disease.

Deep/clinical remission was defined as complete mucosal healing and CDAI score <150.

Sum of treatments exceeds 100% to allow for combination treatments.

No differences were seen in age, gender, body mass index (BMI), or disease duration in CD patients with different levels of disease activity. Ethnicity also showed no significant difference between groups, where White/Caucasian patients made up the vast majority of the sample (Table 1). Discordance was found between CDAI score and physician-reported remission; of the 39 patients with a CDAI >220 (therefore objectively classified as having moderate-to-severe disease activity), 10 (25.6%) were actually reported to be in remission by their physician. However, remission status in the physicians’ subjective opinion was significantly different across disease activity groups (*P* < .0001), with objectively greater disease activity associated with a reduction in the proportion of patients reported by their physician to be in remission (Table 1). The majority of patients were considered by their physician to be currently stable, but there was a nonsignificant trend for a decreasing proportion of stable patients, and an increasing proportion of deteriorating patients, with increasing disease activity (Table 1). The proportion of patients having a flare, and the number of flares per patient in the past 12 months increased with increasing disease activity (both *P* < .0001, Table 1), as did the proportion of patients who were experiencing a flare at the time of data collection (*P* < .01, Table 1). On average, patients with mildly active disease experienced one more flare than patients in remission, and patients with moderately-to-severely active disease experienced 2 more flares than patients in remission in the 12 months prior to data collection.

#### Ulcerative colitis

A total of 282 patients with UC who had matched physician- and patient-reported questionnaires available, who were working, and who had completed the WPAI questionnaire, were included in the primary analyses (Figure 1). Disease activity groups based on current pMayo score showed 83 (29.4%) patients were in remission, 134 (47.5%) had mildly active disease, and 65 (23.0%) had moderately-to-severely active disease (Table 2). Of patients classified as having moderately-to-severely active disease, the median pMayo score was 5, and only 9 patients had a pMayo score >6.

**Table 2.** Demographics, clinical characteristics, and current treatments by pMayo score-defined disease activity in patients with UC.

	Overall (*n* = 282)	Remission^a^ (*n* = 83)	Mild^a^ (*n* = 134)	Moderate-to-severe^a^ (*n* = 65)	*P*
Age, years					
*n*	282	83	134	65	
Mean (SD)	40.5 (12.1)	42.0 (13.1)	39.8 (11.5)	40.2 (11.8)	.5547
Range	22, 70	22, 70	22, 67	22, 62	
Gender, *n* (%)					
*n*	282	83	134	65	.0368
Female	125 (44.3)	28 (33.7)	69 (51.5)	28 (43.1)	
BMI, kg m^−2^					
*n*	282	83	134	65	
Mean (SD)	25.5 (3.9)	26.4 (4.6)	25.0 (3.4)	25.5 (3.7)	.2130
Range	17.5, 47.7	19.0, 47.7	17.5, 32.4	17.8, 34.9	
pMayo score					
*n*	282	83	134	65	
Mean (SD)	2.8 (2.0)	0.4 (0.5)	2.8 (0.8)	5.7 (0.9)	<.0001
Median	3	0	3	5	
Range	0, 8	0, 1	2, 4	5, 8	
Disease duration, years					
*n*	249	71	121	57	
Mean (SD)	5.4 (6.0)	7.3 (7.2)	5.1 (5.6)	3.7 (4.3)	.0007
Range	0, 37	0.1, 30	0, 37	0, 15	
Physician-reported patient ethnicity, *n* (%)					
*n*	282	83	134	65	
White/Caucasian	230 (81.6)	66 (79.5)	112 (83.6)	52 (80.0)	
Asian-Indian subcontinent	2 (0.7)	1 (1.2)	0 (0.0)	1 (1.5)	
Asian other	5 (1.8)	2 (2.4)	3 (2.2)	0 (0.0)	
Hispanic/Latino	9 (3.2)	3 (3.6)	5 (3.7)	1 (1.5)	.3133
Middle Eastern	4 (1.4)	3 (3.6)	1 (0.7)	0 (0.0)	
Mixed race	1 (0.4)	0 (0.0)	0 (0.0)	1 (1.5)	
African American	30 (10.6)	7 (8.4)	13 (9.7)	10 (15.4)	
Other	1 (0.4)	1 (1.2)	0 (0.0)	0 (0.0)	
Physician-reported current remission status^b^, *n* (%)					
*n*	282	83	134	65	
Not in remission	123 (43.6)	7 (8.4)	60 (44.8)	56 (86.2)	
In remission	138 (48.9)	63 (75.9)	66 (49.3)	9 (13.8)	<.0001
In deep/clinical remission	21 (7.4)	13 (15.7)	8 (6.0)	0 (0.0)	
Physician-reported current disease progression, *n* (%)					
*n*	282	83	134	65	
Improving	83 (29.4)	13 (15.7)	48 (35.8)	22 (33.8)	
Stable	167 (59.2)	70 (84.3)	81 (60.4)	16 (24.6)	<.0007
Deteriorating	32 (11.3)	0 (0.0)	5 (3.7)	27 (41.5)	
Physician reported: patient currently experiencing a flare, *n* (%)					
*n*	282	80	122	56	
Yes	42 (16.3)	1 (1.3)	10 (8.2)	31 (55.4)	<.0001
Physician reported: patient flared in past 12 months, *n* (%)					
*n*	258	80	122	56	
Yes	147 (57.0)	22 (27.5)	74 (60.7)	51 (91.1)	<.0001
Physician reported: number of flares/patient in the last 12 months					
*n*	258	80	122	56	
Mean (SD)	1.2 (2.6)	0.4 (0.7)	1.4 (3.1)	2.0 (2.8)	<.0001
Range	0, 30	0, 3	0, 30	0, 20	
Current treatment^c^, *n* (%)					
*n*	274	79	130	65	
Biologic	110 (40.1)	30 (38.0)	53 (40.8)	27 (41.5)	.8921
5-ASA	184 (67.2)	53 (67.1)	86 (66.2)	45 (69.2)	.9111
Immunomodulator	91 (33.2)	25 (31.6)	35 (26.9)	31 (47.7)	.0139
Corticosteroid	84 (30.7)	4 (5.1)	41 (31.5)	39 (60.0)	<.0001
Patient-reported satisfaction with current treatment, *n* (%)					
*n*	272	81	129	62	
Satisfied	206 (75.7)	77 (95.1)	93 (72.1)	36 (58.1)	<.0001
Not satisfied	66 (24.3)	4 (4.9)	36 (27.9)	26 (41.9)	

Abbreviations: 5-ASA, 5-amino salicylic acid; BMI, body mass index; pMayo, partial Mayo; UC, ulcerative colitis; UCDAI, ulcerative colitis disease activity index. *n* values are reported per row for extra clarity, as the different variables used in analyses do not always have the same sample sizes.

pMayo score 0–1, remission; pMayo score 2–4, mild disease; pMayo score >4, moderate-to-severe disease.

Deep/clinical remission was defined as complete mucosal healing and UCDAI sigmoidoscopy score >0.

Sum of treatments exceeds 100% to allow for combination treatments.

A significant association of gender with disease activity was seen for UC patients (*P* < .05, Table 2), with a lower proportion of female patients in remission than with active disease. There was also an association between the number of years since a patient was first diagnosed with UC and disease activity, with activity diminishing with increasing disease duration (*P* < .001, Table 2). There was no association with ethnicity and disease severity, again with White/Caucasian patients making up the majority of the sample (Table 2). Discordance was found between pMayo score and physician-reported remission; of the 65 patients with a pMayo score >4 (therefore objectively classified as having moderate-to-severe disease activity), 9 (13.8%) were actually reported to be in remission by their physician. However, physician-reported remission status was significantly different across UC disease activity groups (*P* < .0001) with patients having objectively greater disease activity less likely to be considered to be in remission by their physician (Table 2). Compared with patients in remission, patients with more active disease were more likely to be deteriorating, to have flared in the past 12 months, to have experienced more flares in the past 12 months, and to be experiencing a flare at the time of data collection (all *P* < .001, Table 2). On average, patients with mildly active disease experienced one more flare than patients in remission, and patients with moderately-to-severely active disease experienced 1.6 more flares than patients in remission in the 12 months prior to data collection.

#### Demographics and clinical characteristics by working status

The demographics and clinical characteristics of the 459 CD patients and 600 UC patients with matched physician- and patient-reported questionnaires, data available to calculate disease activity, and known working status (Figure 1) are reported by working status in Supplementary Tables S1 and S2. A significant difference was noted in objective disease activity groupings between working and nonworking CD (*P* < .01) and UC (*P* < .05) patients. In CD patients, 66.1% and 52.7% of working and nonworking patients, respectively, were classified as in remission, and in UC patients, 26.6% and 21.4% of working and nonworking patients, respectively, were classified as in remission.

### Current Treatment

In CD, the most common class of current treatment was biologics, followed by 5-ASA (Table 1). No significant difference was seen in current treatment between CD patients in different disease activity groups, with the exception of corticosteroids, which were more commonly prescribed for patients with active CD than those in remission (*P* < .0001, Table 1). The proportion of patients reporting that they were satisfied with current treatment decreased with increasing disease activity in patients with CD (*P* < .0001, Table 1).

Overall, the most common class of current treatment in patients with UC was 5-ASA, followed by biologics, with no significant difference between patients in different disease activity groups in the use of these classes of treatment (Table 2). However, the proportions of patients receiving immunomodulators (*P* < .05) and corticosteroids (*P* < .0001) varied based on disease activity, with the highest proportions of patients receiving both of these classes of drugs being those with moderate-to-severe UC (Table 2). The proportion of patients reporting that they were satisfied with current treatment decreased with increasing disease activity in patients with UC (*P* < .0001, Table 2).

#### Treatment satisfaction by working status

Both CD and UC patients who were working were more likely to be satisfied with current treatment than those who were not working (Supplementary Tables S1 and S2). Of working CD and UC patients, 72.8% and 78.7%, respectively, reported being satisfied, compared with 58.5% and 69.1%, respectively, of those who were not working (*P* < .01).

### Impaired Productivity

**Figure 2.**
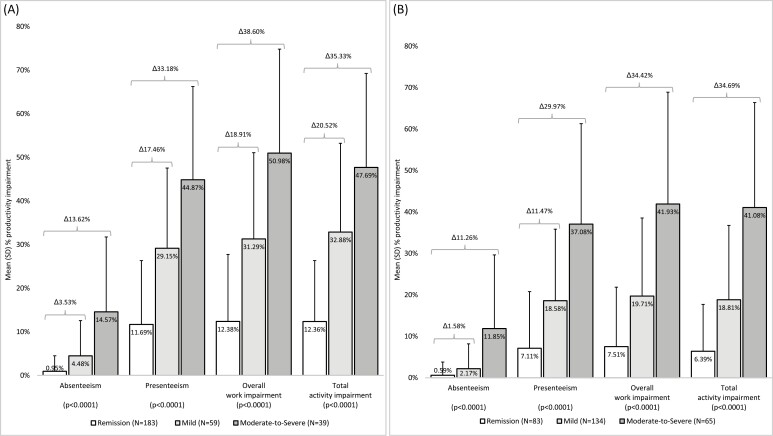
Productivity impairment by disease activity. (A) Patients with CD and (B) patients with UC. Δ Percentage points difference; #Total activity impairment, *n* = 182. Abbreviations: CD, Crohn’s disease; UC, ulcerative colitis.

Based on WPAI scores, both CD and UC had an impact on productivity at work, with overall work impairment around 50% in patients with moderate-to-severe CD and just over 40% in patients with moderate-to-severe UC (Figure 2). Similar levels of impairment in daily activities outside work were reported by patients with moderately-to-severely active CD and UC (Figure 2). Presenteeism was 45% and 37% for patients with moderate-to-severe CD and UC, respectively (Figure 2). Absenteeism also contributed to work productivity loss, but to a lesser extent than presenteeism, with absenteeism reported as 15% and 12% for patients with moderately-to-severely active CD and UC, respectively (Figure 2). Greater productivity impairment was observed with greater CD and UC disease activity for all measures of productivity reported in the WPAI (absenteeism, presenteeism, overall work impairment, and total activity impairment outside of work) (all *P* < .0001, Figure 2).

### Indirect Cost Due to Impaired Productivity

**Figure 3.**
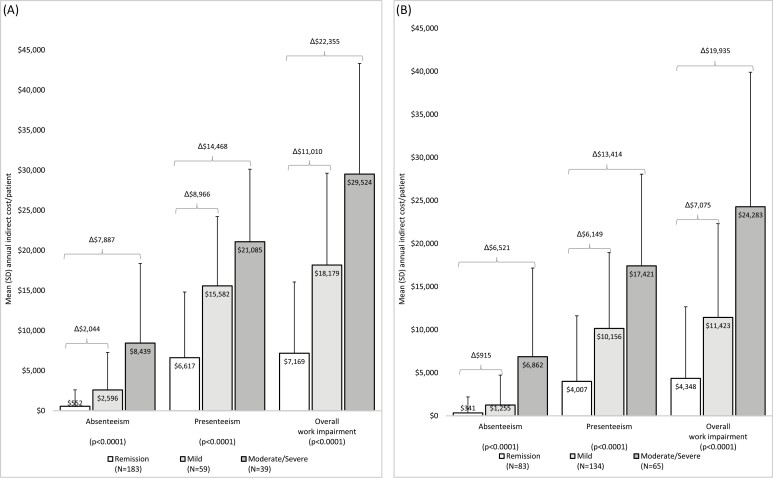
Annual indirect costs due to absenteeism, presenteeism, and overall work productivity impairment by disease activity. (A) Patients with CD and (B) patients with UC. Abbreviations: CD, Crohn’s disease; UC, ulcerative colitis.

The mean annual indirect costs per patient associated with CD and UC (calculated based on impairment in absenteeism, presenteeism, and overall work impairment reported above) increased with increasing disease activity (all *P* < .0001, Figure 3). Indirect costs calculated based on overall work impairment were $29 524/patient/year for patients with moderately-to-severely active CD and $24 283/patient/year for patients with moderately-to-severely active UC (Figure 3).

## Discussion

This analysis of data derived from a large, real-world survey of gastroenterologists and their consulting patients with IBD in the United States adds to existing literature examining the impact of CD and UC on work-related productivity loss and associated indirect costs. This study reported productivity impairment and associated costs for patients with CD or UC by disease activity health state, and showed that patients with CD and UC experienced increased absenteeism and presenteeism with worsening disease activity, resulting in higher indirect economic costs.

Impaired productivity and associated costs were reported by patients with both CD and UC, with reductions mainly through reduced productivity while at work (presenteeism) rather than missed work time (absenteeism). Some degree of impairment was observed even in patients objectively assessed as being in remission, but there was a significant association of greater productivity impairment with higher disease activity.

Previously published studies have described reduced productivity in patients with IBD, with productivity levels decreasing with increasing disease activity, although a range of impairment levels have been reported. Data from a US tertiary IBD center using the WPAI showed presenteeism for CD and UC to be 61% and 64%, respectively, and absenteeism to be 20% and 22%, respectively.^14^ Our analysis showed presenteeism for CD and UC patients to be 29% and 19%, respectively, in mildly active disease and 45% and 37%, respectively, in moderately-to-severely active disease; absenteeism in our study was 4% and 2%, respectively, in mildly active disease, and 15% and 12%, respectively, in moderately-to-severely active disease. The published study did not stratify patients by disease activity level. The greater level of impairment in the published study compared to patients with active disease in our analysis might result from the tertiary IBD center setting. A high proportion of patients receiving care from a tertiary care center would be expected to have severely active disease, while the majority of patients in our study classified as having moderately-to-severely active disease had moderately active disease. Our study, however, was in agreement with the finding from the US tertiary IBD center-based study in that presenteeism accounts for the majority of indirect costs.^14^

In a multicenter observational study in Brazil, patients with moderately-to-severely active CD (CDAI score >220) reported median absenteeism, presenteeism, overall work impairment, and total activity impairment of 5%, 10%, 30%, and 50%, respectively, while those patients with mildly active or no CD disease activity reported 0%, 10%, 20%, and 20%, respectively.^28^ This study also reported median absenteeism, presenteeism, overall work impairment, and total activity impairment of 6%, 20%, 35%, and 75%, respectively, in patients with moderately-to-severely active UC (pMayo score >4), but 0%, 0%, 0%, and 10% in patients with mildly active or no UC disease activity.^28^ Our study generally found greater levels of impairment for patients with moderately-to-severely active disease than those observed in the published Brazilian study, using the same definitions of moderately-to-severely active disease. The discrepancies may be explained, at least in part, by the smaller sample sizes of working patients included in the analysis of productivity in the Brazilian study compared with our study (111 and 58 patients with CD and UC, respectively, in the Brazilian study, compared with 281 and 282 patients with CD and UC, respectively, in our study). The Brazilian study did not report numbers of patients included in the productivity analysis by disease activity.

An Australian multicenter observational study found mean absenteeism, presenteeism, overall work impairment, and total activity impairment in patients with moderately-to-severely active UC (pMayo score >4) of 43%, 37%, 48%, and 52%; these findings are comparable with those we report here for UC patients with moderately-to-severely active disease, with the exception of absenteeism, which we reported to be only 12%. In our study, the level of productivity impairment based on each of these parameters was greater in patients with moderate-to-severe UC than those in remission or with mild disease, but in the Australian study, the trend for increasing impairment with increasing disease activity was seen only for total activity impairment.^29^

As current treatment options in IBD provide the potential for reduced disease activity and achievement of remission, it is important to consider the benefit of such changes to patients and to society as a whole. Our results clearly demonstrated the statistical association of greater disease activity with greater impairment of productivity in both CD and UC, but the clinical significance of these findings should also be considered. The minimal clinically important difference (MCID) on the WPAI in CD has been estimated to be 6.5% for absenteeism, 6.1% for presenteeism, 7.3% for overall work impairment, and 8.5% for total activity impairment.^30^ Based on these figures, we observed clinically meaningful differences in all productivity impairment parameters between the 3 disease activity levels defined in our study, with the exception of the difference between patients in remission and those with mildly active CD for absenteeism. No published MCIDs are available for the WPAI in UC, but if the threshold reported for CD are applied, clinically meaningful differences were also observed in UC patients for all WPAI endpoints between patients in remission versus those with mild disease, as well as patients with mild disease versus those with moderate-to-severe disease, with the exception of absenteeism between patients in remission versus those with mild UC disease activity.

Our study showed indirect costs due to absenteeism and presenteeism in both CD and UC, both of which increased significantly with increasing disease activity. The cost related to absenteeism in CD ranged from $552 to $8439/patient/year, which is generally consistent with the $2819 estimated for a sample of patients with CD with all levels of disease activity in an analysis of US claims data.^11^ However, unlike the direct elicitation of absenteeism data from patients in our study, absenteeism was imputed in the published analysis of claims data.^11^ The cost related to absenteeism in an analysis of data from a US survey of patients with IBD was reported to be $783/patient/year, but these data were collected from 1996 to 2006 and the findings are therefore somewhat dated.^15^ In UC patients, we observed absenteeism-related costs of $341–$6862/patient/year, depending on disease activity level, which again is generally consistent with the cost of $2592 reported for a UC sample with all disease activity levels from another US claims analysis (although this published study also used imputed absenteeism data).^12^

In our study, presenteeism-related indirect costs were $6617–$21 085/patient/year and $4007–$17 421/patient/year across disease activity levels in CD and UC, respectively. Previously, there has been limited information available on indirect costs related to presenteeism in IBD, and we have been unable to identify studies reporting the impact of disease activity levels on presenteeism-related costs. Due to the methodological limitations of claims analysis studies, presenteeism-related indirect costs were not able to be estimated in the published US claim analyses,^11,12^ while our survey collected information on presenteeism directly from the participating patients. Our finding that presenteeism was the greatest contributor to work productivity loss and associated indirect costs in IBD aligns with findings from the published analysis of data from a tertiary IBD center, although this published study included only 140 patients with CD and 143 with UC.^14^

A published systematic review of international studies including patients with varying levels of disease activity reported annual indirect cost per patient for lost productivity in IBD to be $14 136 in CD and $6583 in UC.^31^ These figures fall within the ranges we reported from our analysis of indirect costs of IBD resulting from overall work impairment: $7169/patient/year for patients with CD in remission to $29 524/patient/year for patients with moderately-to-severely active CD, and $4348/patient/year for patients with UC in remission to $24 283/patient/year for patients with moderately-to-severely active UC. The costs associated with overall work impairment that we calculated for patients in remission were somewhat lower than the $17 766 reported from a prospective US study for patients with IBD in remission.^14^

Our findings show that if low disease activity, and preferably remission, is achieved in IBD, there is a potential for reduced indirect costs.

A number of potential limitations should be considered in the evaluation of our findings. This analysis was based on a convenience sample, and care should be taken in generalizing findings to a broader patient population. The DSP did not include a true random sample of physicians or patients; while minimal inclusion criteria governed the selection of the participating physicians, participation was influenced by willingness to complete the survey. Physicians were asked to provide data for a consecutive series of patients to avoid selection bias, but no formal patient selection verification procedures were followed. Identification of the target patient group was based on the judgment of the respondent physician and not a formalized diagnostic checklist but was representative of physicians’ real-world classification of patients. Patients participating in the survey had to visit their physician during the survey period, and thus may reflect patients who consult more frequently than the general IBD population. However, as previously highlighted, there were few patients with severely active disease included in our analysis population, which might reflect the fact that patients who were hospitalized or whose disease was too severe for them to go to a consultation were not included in our study. The point-in-time design of this study prevents any conclusions about causal relationships; however, identification of significant associations is possible. Recall bias might have affected the responses of both patients and physicians to the questionnaires, which is a common limitation of surveys. However, the data for these analyses were collected at the time of each patient’s appointment and this is expected to reduce the likelihood of recall bias. Moreover, the physician is asked to complete the forms using the patient chart records, to further reduce the likelihood of recall bias. While our research design included methods to ensure that physicians and staff were unaware of patient responses on the patient-reported forms, it was not possible to confirm that no information exchange occurred between physicians and their patients. This has the potential to influence patient responses to the standardized patient-reported outcome questionnaires. Another potential limitation is that comparing patient data between community hospitals and tertiary care center’s may not be a completely equal comparison, which is something that was not accounted for in this study. It could be considered for future studies, however data and conclusions here should be interpreted with caution.

Despite such limitations, real-world studies play an important part in highlighting areas of concern that are not addressed in clinical trials. Patients included in clinical trials represent a small proportion of the consulting population due to age restrictions and stringent eligibility criteria.^32^ As a result, data from real-world studies can complement clinical trials and provide insight into interventions and outcomes commonly seen in clinical practice.

## Conclusion

In conclusion, clear associations of disease activity in CD and UC with productivity loss and associated indirect costs were demonstrated in our analysis. As far as we are aware, this is the first study to show increasing presenteeism impairment and associated indirect costs with increasing disease activity. We also observed higher disease activity and lower treatment satisfaction in IBD patients who were not working compared with those working. Although the methodological approach we adopted precludes conclusions on causality for these observations, they do suggest that novel IBD treatments able to achieve significant reductions in disease activity could provide meaningful improvements in humanistic and economic outcomes, as well as clinical outcomes.

## Supplementary Material

otac023_suppl_Supplementary_Table_S1

otac023_suppl_Supplementary_Table_S2

## Data Availability

Data not publicly available.
