# Supplementary material for: Work-Related Productivity Loss and Associated Indirect Costs in Patients With Crohn’s Disease or Ulcerative Colitis in the United States
Source: Crohns Colitis 360. 2022 Jun 15;4(3):otac023. doi: 10.1093/crocol/otac023 (PMC9802455; doi:10.1093/crocol/otac023)
Supplement: otac023_suppl_Supplementary_Table_S1 [file otac023_suppl_supplementary_table_s1.docx]

**Table S1.** Demographics and clinical characteristics by working status in patients with CD

|  | **Overall**  **(n=459)** | **Not working**  **(n=146)** | **Working**  **(n=313)** | **p-value** |
| --- | --- | --- | --- | --- |
| **Age, years** |  |  |  |  |
| n  Mean (SD)  Range | 459  40.5 (15.4)  18, 85 | 146  41.5 (20.9)  18, 85 | 313  40.0 (12.0)  20, 68 | 0.2087 |
| **Gender, n (%)**  n  Female | 459  252 (54.9) | 146  100 (68.5) | 313  152 (48.6) | <0.0001 |
| **BMI, kg/m^2^** |  |  |  |  |
| n  Mean (SD)  Range | 459  25.3 (4.7)  15.9, 68.3 | 146  24.6 (5.8)  15.9, 68.3 | 313  25.6 (4.1)  16.2, 46.9 | 0.0019 |
| **Employment status, n (%)** |  |  |  |  |
| n  Working full-time  Working part-time  On long-term sick leave  Homemaker  Student  Retired  Unemployed | 459  261 (56.9)  52 (11.3)  5 (1.1)  26 (5.7)  54 (11.8)  34 (7.4)  27 (5.9) | 146  0 (0.0)  0 (0.0)  5 (3.4)  26 (17.8)  54 (37.0)  34 (23.3)  27 (18.5) | 313  261 (83.4)  52 (16.6)  0 (0.0)  0 (0.0)  0 (0.0)  0 (0.0)  0 (0.0) | <0.0001 |
| **Disease duration, years** |  |  |  |  |
| n | 409 | 128 | 281 |  |
| Mean (SD)  Range | 7.0 (7.8)  0, 45 | 6.6 (8.5)  0, 45 | 7.1 (7.4)  0, 40 | 0.0463 |
| **Current disease activity based on CDAI^a^, n (%)** |  |  |  |  |
| n  Remission | 459  284 (62.9) | 146  77 (52.7) | 313  207 (66.1) |  |
| Mild | 104 (22.7) | 39 (26.7) | 65 (20.8) | 0.0026 |
| Moderate/Severe | 71 (15.5) | 30 (20.5) | 41 (13.1) |  |
| **Physician-reported current remission status**^b^**, n (%)** |  |  |  |  |
| n  Not in remission | 459  197 (42.9) | 146  72 (49.3) | 313  125 (39.9) |  |
| In remission | 223 (48.6) | 65 (44.5) | 158 (50.5) | 0.1263 |
| In deep/clinical remission | 39 (8.5) | 9 (6.2) | 30 (9.6) |  |
| **Physician-reported current disease progression, n (%)** |  |  |  |  |
| n  Improving | 459  147 (32.0) | 146  53 (36.3) | 313  94 (30.0) |  |
| Stable | 262 (57.1) | 66 (45.2) | 196 (62.6) | 0.6974 |
| Deteriorating | 50 (10.9) | 27 (18.5) | 23 (7.3) |  |
| **Physician-reported: Patient currently experiencing a flare, n (%)** |  |  |  |  |
| n | 407 | 124 | 283 |  |
| Yes | 58 (14.3) | 25 (20.2) | 33 (11.7) | 0.0306 |
| **Physician-reported: Patient flared in past 12 months, n (%)** |  |  |  |  |
| n | 407 | 124 | 283 |  |
| Yes | 229 (56.3) | 75 (60.5) | 154 (54.4) | 0.2786 |
| **Physician-reported: Number of flares in the past 12 months** |  |  |  |  |
| n | 407 | 124 | 283 |  |
| Mean (SD)  Range | 1.2 (2.4)  0, 30 | 1.5 (3.3)  0, 30 | - 1. (1.8)   2. 0, 16 | 0.2115 |
| **Patient-reported satisfaction with current treatment, n (%)** |  |  |  |  |
| n | 443 | 142 | 301 |  |
| Satisfied | 302 (68.2) | 83 (58.5) | 219 (72.8) | 0.0014 |
| Not satisfied | 141 (31.8) | 59 (41.5) | 82 (27.2) |  |

^a^CDAI score 0‒150, remission; CDAI score 151‒220, mild disease; CDAI score >220, moderate-to-severe disease

^b^deep/clinical remission was defined as complete mucosal healing and a CDAI score <150 for patients with CD or a UCDAI sigmoidoscopy score >0 for patients with UC

BMI, body mass index; CD, Crohn’s disease; SD, standard deviation

n values are reported per row for extra clarity, as the different variables used in analyses do not always have the same sample sizes
