# Supplementary material for: Work-Related Productivity Loss and Associated Indirect Costs in Patients With Crohn’s Disease or Ulcerative Colitis in the United States
Source: Crohns Colitis 360. 2022 Jun 15;4(3):otac023. doi: 10.1093/crocol/otac023 (PMC9802455; doi:10.1093/crocol/otac023)
Supplement: otac023_suppl_Supplementary_Table_S2 [file otac023_suppl_supplementary_table_s2.docx]

**Table S2.** Demographics and clinical characteristics by working status in patients with UC

|  | **Overall**  **(n=600)** | **Not working**  **(n=182)** | **Working**  **(n=418)** | **p-value** |
| --- | --- | --- | --- | --- |
| **Age, years** |  |  |  |  |
| n  Mean (SD)  Range | 600  42.5 (15.6)  18, 90 | 182  45.4 (21.3)  18, 90 | 418  41.2 (12.0)  19, 78 | 0.2253 |
| **Gender, n (%)**  n  Female | 600  305 (50.8) | 182  116 (63.7) | 418  189 (45.2) | <0.0001 |
| **BMI, kg/m^2^** |  |  |  |  |
| n  Mean (SD)  Range | 600  26.2 (4.5)  16.0, 61.0 | 182  25.9 (4.6)  16.0, 43.4 | 418  26.3 (4.5)  17.2, 61.0 | 0.3734 |
| **Employment status, n (%)** |  |  |  |  |
| n  Working full-time  Working part-time  On long-term sick leave  Homemaker  Student  Retired  Unemployed | 600  360 (60.0)  58 (9.7)  2 (0.3)  41 (6.8)  60 (10.0)  61 (10.2)  18 (3.0) | 182  0 (0.0)  0 (0.0)  2 (1.1)  41 (22.5)  60 (33.0)  61 (33.5)  18 (9.9) | 418  360 (86.1)  58 (13.9)  0 (0.0)  0 (0.0)  0 (0.0)  0 (0.0)  0 (0.0) | <0.0001 |
| **Disease duration, years** |  |  |  |  |
| n | 527 | 168 | 359 |  |
| Mean (SD)  Range | 5.8 (7.1)  0, 44 | 6.4 (8.8)  0, 44 | 5.5 (6.1)  0, 37 | 0.4643 |
| **Current disease activity based on pMayo score^a^, n (%)** |  |  |  |  |
| n  Remission | 600  150 (25.0) | 182  39 (21.4) | 418  111 (26.6) |  |
| Mild | 297 (49.5) | 86 (47.3) | 211 (50.5) | 0.0298 |
| Moderate-to-severe | 153 (25.5) | 57 (31.3) | 96 (23.0) |  |
| **Physician-reported current remission status**^b^**, n (%)** |  |  |  |  |
| n  Not in remission | 600  292 (48.7) | 182  99 (54.4) | 418  193 (46.2) |  |
| In remission | 261 (43.5) | 69 (37.9) | 192 (45.9) | 0.1617 |
| In deep/clinical remission | 47 (7.8) | 14 (7.7) | 33 (7.9) |  |
| **Physician-reported current disease progression, n (%)** |  |  |  |  |
| n  Improving | 600  200 (33.3) | 182  69 (37.9) | 418  131 (31.3) |  |
| Stable | 326 (54.3) | 80 (44.0) | 246 (58.9) | 0.9630 |
| Deteriorating | 74 (12.3) | 33 (18.1) | 41 (9.8) |  |
| **Physician-reported: Patient currently experiencing a flare,  n (%)** |  |  |  |  |
| n | 553 | 169 | 384 |  |
| Yes | 92 (16.6) | 32 (18.9) | 60 (15.6) | 0.3855 |
| **Physician-reported: Patient flared in past 12 months, n (%)** |  |  |  |  |
| n | 553 | 169 | 384 |  |
| Yes | 286 (51.7) | 95 (56.2) | 101 (49.7) | 0.1670 |
| **Physician-reported: Number of flares in the past 12 months** |  |  |  |  |
| n | 553 | 169 | 384 |  |
| Mean (SD)  Range | 1.1 (2.2)  0, 30 | 1.2 (2.7)  0, 30 | 1.0 (1.9)  0, 20 | 0.1436 |
| **Patient-reported satisfaction with current treatment, n (%)** |  |  |  |  |
| n | 570 | 175 | 395 |  |
| Satisfied | 432 (75.8) | 121 (69.1) | 311 (78.7) | 0.0099 |
| Not satisfied | 138 (24.2) | 54 (30.9) | 84 (21.3) |  |

^a^pMayo score 0‒1, remission; pMayo score 2‒4, mild disease; pMayo score >4, moderate-to-severe disease

^b^In the physician’s opinion; deep/clinical remission was defined as complete mucosal healing and a CDAI score <150 for patients with CD or a UCDAI sigmoidoscopy score >0 for patients with UC

BMI, body mass index; pMayo, partial Mayo, SD, standard deviation; UC, ulcerative colitis; UC, ulcerative colitis disease activity index

n values are reported per row for extra clarity, as the different variables used in analyses do not always have the same sample sizes
